# Processing, Characterization and Applications of Ceramic Matrix Composites

**DOI:** 10.3390/ma19122480

**Published:** 2026-06-10

**Authors:** Rodrigo Moreno, Oscar Rubem Klegues Montedo

**Affiliations:** 1 Institute of Ceramics and Glass (ICV), CSIC, 28049 Madrid, Spain; 2 Grupo de Biomateriais e Materiais Nanoestruturados, Laboratório de Cerâmica Técnica (CerTec), Programa de Pós-graduação em Ciência e Engenharia de Materiais, Universidade do Extremo Sul Catarinense (UNESC), Criciúma 88806-000, Brazil; okm@unesc.net

## 1. Introduction

The increasing demand for new products and the development of new technologies have inspired a search for novel materials that are able to satisfy the increasing exigencies of higher resistance against aggressive environments, enhanced thermomechanical properties, finer and better controlled microstructures and improved reliability and durability in service [1,2,3]. Hybrid materials have demonstrated increasing feasibility in a growing range of applications. In particular, the design and manufacture of advanced ceramic matrix composites play prominent roles in the integration of components and technologies operating under extreme thermal conditions or aggressive environments [4,5,6,7,8]. Ceramic matrices are well adapted to those hard solicitations in service, while the secondary phases allow the material’s performance to be tailored by adding precise functionalities or enhancing the final properties, focusing on a longer mean life [9] and a suitable transition to a circular economy based on sustainability and low environmental impact [10].

The Special Issue “Processing, Characterization and Applications of Ceramic Matrix Composites” presents the most recent advances and the latest trends and novel developments in the design, manufacture, and characterization of ceramic matrix hybrid materials, encompassing both the fundamentals and the applied technology.

## 2. Brief Description of Published Articles

This Special Issue comprises 12 published papers (ten full-length articles and two reviews) covering a range of topics representative of existing research on ceramic matrix composites. Medvedovski and Travitzky (Contribution 1) reviewed the state of the art of the additive manufacturing of functionally graded ceramics for armor applications and demonstrated the feasibility of the FGM design for enhancing ballistic performance and reducing weight. Meanwhile, in their review, Gao et al. (Contribution 2) focused on C/C composites that have been modified with ultra-high-temperature ceramics to improve their oxidation and ablation resistance, considering their applications, processing, performance, structural design, microstructure, etc.

In addition to those two great review papers, this Special Issue contains ten full-length articles devoted to different aspects of the manufacturing process and characterization of several composite materials, including ultra-high-temperature ceramics, Si_3_N_4_/BN composites, laminated Sc_2_SnC MAX coatings on SiC fibers, mortars, alumina/eucryptite nanostructure materials, diopside glass ceramics, networks infiltrated with polymers, magnetic CuFe_2_O_4_ spinel–polypyrrole composites, and carbon nanotube–YSZ nanocomposites.

Ultra-high-temperature ceramic matrix composites (UHTCMCs) can be used as structural components, providing useful properties such as high temperature performance, low density and damage tolerance [11]. Jindai et al. (Contribution 3) developed a probabilistic model to evaluate thermal shock sensitivity and early-stage fatigue behavior in fiber-reinforced UHTCMC thrusters, with the aim of reducing costs and the risk of damage. The same authors (Contribution 4) performed a finite element investigation of the design optimization of a UHTCMC thruster for use in green propulsion systems. Further research is needed to validate the proposed numerical models.

Several studies reported the densification, the mechanical properties and the thermal shock resistance of Si_3_N_4_/BN composite ceramics [12,13]. Gong et al. (Contribution 5) describe a Si_3_N_4_/BN composite manufactured via a two-step sintering process that combines pseudo-hot isostatic pressing with gas pressure sintering. Its properties can be tailored to control the BN particle size, which determines the density and the texture.

The family of MAX phases is also attracting increased interest. The presence of MAX layers in SiC structures is indicative of their enhanced mechanical properties. The popular Ti-based phases decompose into TiC at high temperature; thus, other compositions must be developed [14]. Wang et al. (Contribution 6) successfully synthesized a Sc_2_SnC coating on the surface of SiC fibers using the molten salt method through the reaction of metals with pyrolytic carbon.

The processing and characterization of oxidic-based ceramic matrix composites were also studied in this SI. The thermomechanical properties of oxides are often enhanced by adding small concentrations of a second phase. Arcaro et al. [15] demonstrated that adding a second phase of nanosized alumina to a glass ceramic matrix facilitated a reduction in the coefficient of thermal expansion (CTE) from 9.5 to 4.4 × 10^−6^ °C^−1^, while Inocente et al. [16] synthesized lithium aluminum silicate nanoparticles at 850 °C with a low CTE (1.25 × 10^−6^ °C^−1^). Inocente et al. (Contribution 7) synthesized eucryptite nanoparticles via a heterocoagulation route; these are used as reinforcing phase within an alumina matrix, reducing the sintering temperature by 200 °C whilst also reducing the grain size. Karamanov et al. (Contribution 8) contributed a paper focused on diopside-based glass ceramics. They listed the advantages of using microwave processing in the sinter-crystallization of diopside glass ceramics, revealing a significant reduction in total porosity and the elimination of open porosity. Another study that described the mechanical behavior of oxide ceramic composites was published by Kasperski et al. (Contribution 9), who emphasized the use of non-conventional sintering (spark plasma sintering) in the manufacture of yttria-stabilized zirconia reinforced with double-walled carbon nanotubes, which led to a reduction in the friction coefficients and, therefore, an enhanced wear resistance.

Another group of papers focused on the preparation and performance of CMCs for functional applications. Polymer–ceramic composites are used in many technological fields due to their superior mechanical behavior, thermal response, and chemical inertness [17]. On the other hand, ceramic–polymer composites are used for the development of novel functional ceramics, such as supercapacitors and piezoelectric devices, among others [18]. Award and Zhitomirsky (Contribution 10) investigated the fabrication of ferrimagnetic CuFe_2_O_4_–conductive polypyrrole (PPy) composites for energy storage. The addition of PPy decreased the charge transfer resistance and increased the capacitance. This combination of capacitive and magnetic properties holds potential for novel applications. Wellhäußer et al. (Contribution 11) reported the properties of polymer-infiltrated zinc oxide networks using different acrylate-based polymers. The ZnO network provides hardness; it does not improve strength, but increases long-term stability. Finally, Li et al.’s research (Contribution 12) addressed the control of waterproof, impermeability and water vapor transmission of mortars exposed to three different modifiers and evaluated their effect on the mentioned properties.

## 3. Final Remarks

In summary, the present SI provides an excellent overview of the recent trends in ceramic matrix composites through several review articles summarizing the state of the art in this field. It also presents several original studies focusing on relevant areas of CMC research, including its structural applications, using UHTCMCs, MAX phases, ceramic oxides, carbon nanotubes and glass ceramics as examples, and describes some recent investigations of the use of CMCs in functional applications, including ferrimagnetic devices, polymer-infiltrated ZnO networks and mortars with improved resistance to water attack. The included papers demonstrate the significance of ceramic matrix composites in the development of advanced technologies which require a combination of multiple properties and designs for the manufacture of structural and functional components operating under extreme conditions with increased efficiency and lower environmental impact. The research groups proceeding from different countries covering three continents and a variety of material families, confirming the global interest in and applicability of the topic.
